# A Prospective Phase II Study of Simultaneous Modulated Accelerated Radiotherapy Concurrently With CDDP/S1 for Esophageal Squamous Cell Carcinoma in the Elderly

**DOI:** 10.3389/fonc.2021.760631

**Published:** 2021-11-25

**Authors:** SuPing Guo, FangJie Liu, Hui Liu, YingJia Wu, XuHui Zhang, WenFeng Ye, GuangYu Luo, QiWen Li, NaiBin Chen, Nan Hu, Bin Wang, Jun Zhang, MaoSheng Lin, HuiXia Feng, Bo Qiu

**Affiliations:** ^1^ Department of Radiation Oncology, Sun Yat-Sen University Cancer Center, Guangzhou, China; ^2^ State Key Laboratory of Oncology in South China, Guangzhou, China; ^3^ Collaborative Innovation Center for Cancer Medicine, Guangzhou, China; ^4^ Guangdong Association Study of Thoracic Oncology, Guangzhou, China; ^5^ Department of Clinical Nutrition, Sun Yat-Sen University Cancer Center, Guangzhou, China; ^6^ Department of Endoscopy, Sun Yat-Sen University Cancer Center, Guangzhou, China

**Keywords:** chemoradiotherapy, esophageal cancer, elderly patients, treatment-related toxicity, survival outcome

## Abstract

**Background:**

To explore the efficacy and toxicity of simultaneous modulated accelerated radiotherapy (SMART) concurrently with cisplatin (CDDP) and S1 (tegafur/gimeracil/oteracil) in elderly patients with esophageal squamous cell carcinoma (ESCC).

**Methods:**

This single-arm, phase II study enrolled pathologically confirmed, stage II–IVa ESCC of 70–80 years old and Eastern Cooperative Oncology Group performance status (ECOG PS) 0–2. Patients received SMART (64 Gy to gross tumor volume and 48 Gy to clinical target volume in 30 fractions) with concurrent CDDP (day 1 of each week) and S1 (days 1–14, 22–35). The primary endpoint was objective response rate (ORR). The secondary endpoints included progression-free survival (PFS), overall survival (OS) and toxicities.

**Results:**

Thirty-seven eligible patients were analyzed with median follow-up of 25.7 months for all and 46.1 months for survivors. The ORR was 88.9%. Patients with baseline weight loss <5% (p=0.050) and nutritional risk index (NRI) ≥105.2 (p=0.023) had better tumor response. Median PFS was 13.8 months with 2-year PFS of 37.5%. Median OS was 27.7 months with 2-year OS of 57.5%. OS was significantly associated with ECOG PS (p=0.005), stage (p=0.014), gross tumor volume (p=0.004), baseline NRI (p=0.036), baseline C-reactive protein (CRP) level (p=0.003) and tumor response (p=0.000). CRP level (p=0.016) and tumor response (p=0.021) were independently prognostic of OS. ≥grade 3 anemia, neutropenia and thrombocytopenia occurred in 2.7%, 10.8% and 13.5% of patients; ≥grade 3 esophagitis and pneumonitis occurred in 18.9% and 2.7% of patient, respectively.

**Conclusion:**

SMART concurrently with CDDP/S1 yielded satisfactory response rate, survival outcome and tolerable treatment-related toxicities in elderly patients with ESCC. Further studies are warranted to validate the results.

## Introduction

Esophageal cancer is the sixth leading cause of cancer death worldwide (1). Approximately 30% of patients diagnosed as esophageal cancer are over 70 years’ old (2), so there is an urgent need to optimize the treatment strategy in elderly. Although RTOG8501 has established the role of concurrent chemoradiotherapy (CCRT) in locally advanced esophageal cancer, only 23% of the subjects in the clinical trial were over 70 years old (3). Given that the risk of ≥grade 4 side effects was 10% in the concurrent chemoradiotherapy group, significantly higher than that in the radiotherapy alone group, the concurrent treatment mode is more inclined to younger patients with better general conditions. Elderly patients have greater risk of serious treatment-related toxicities due to less physiologic reserve or more comorbidities, therefore are less likely to receive multimodality treatment compared with younger patients (4). The efficacy and tolerance of CCRT for esophageal cancer in the elderly have not been fully studied, with most of the available researches were retrospective studies or prospective studies with small sample sizes (5–8). How to balance treatment efficacy and safety remains a challenging topic.

Tumor response and locoregional control are vital for the relief of tumor-associated symptoms and the improvement of quality of life in elderly patients. Since most of the local failures after radiotherapy occurred in the location of gross tumor volume (GTV), advanced radiation technique might safely improve the local control by increasing the dose to GTV (9). Simultaneous modulated accelerated radiotherapy (SMART) simultaneously delivers a higher dose per fraction to gross tumor and a relatively lower dose to the elective regions. Dosimetry analysis showed that the SMART plan could increase the dose of GTV from 50.4 Gy to 64.8 Gy while keeping a similar dose to the normal tissue compared with IMRT plan (10). Clinical study also supported the efficacy and safety of SMART at a dose of 59.92 Gy to gross tumor and 50.40 Gy to elective regions in 28 fractions concurrently with paclitaxel and nedaplatin for unresectable esophageal cancer (11). Therefore, we hypothesized that SMART can effectively protect normal tissues while increasing the dose of GTV for esophageal cancer, offering an effective and safety choice for elderly patient.

Cisplatin (CDDP)/5-fluorouracil (5-FU) is one of the most common chemotherapy regimens used concurrently with radiotherapy for esophageal cancer. The use of CDDP/5-FU regimen in elderly patients is limited by its high incidence of adverse effects (7). S1 is an oral 5-FU derivate composed of tegafur, gimeracil and oteracil. It also acts as a RT sensitizer. Studies have shown that S1 has superior efficacy and lower risk of toxicities than 5-Fu (12, 13). In clinical studies, RT concurrently with CDDP/S1 achieved promising response rates of 64.4–89.7% with modest toxicities in non-age-selected esophageal cancer (14, 15). Based on its modest toxicities in esophageal cancer, we hypothesized that CDDP/S1 might be a feasible concurrent chemotherapy regimen for elderly patients.

Although SMART and CDDP/S1 showed promising results in esophageal cancer, the evidence in elderly patients is still very limited. Therefore, we carried out this prospective, phase II trial to explore the efficacy and toxicity of SMART concurrently with CDDP/S1 for elderly patients with esophageal squamous cell carcinoma (ESCC).

## Materials and Methods

### Study Design and Participants

This was a single-arm, phase II study. Eligibility criteria included pathologically confirmed ESCC; stage II–IVa (AJCC TNM staging system, 7th edition) confirmed by endoscopic ultrasonography, CT imaging, bone scan and/or PET scan; aging 70 to 80; ECOG performance status of 0–2; Charlson score ≤4; weight loss ≤15% within the past 6 months; forced expiratory volume in 1s≥1L; adequate bone marrow, hepatic and renal functions; and ability to provide informed consent. Patients with prior chemotherapy, radiotherapy or biological therapy were excluded. This study was approved by the review board of our center and conducted according to the Declaration of Helsinki. Written informed consent was obtained from all participants.

### Treatment

Patients were immobilized using a vacuum bag in the supine position, and underwent a planning CT scan with 5-mm-thick slices. Four dimensional CT was performed to account for respiratory motion. GTV was contoured as visible primary tumors and positive lymph nodes based on endoscopy, CT and/or positron emission tomography (PET) scans. Clinical target volume (CTV) included GTV plus a lateral margin of 0.5–1.0 cm, a longitudinal margin of 3–4 cm and elective lymph nodes regions. The planning target volume for GTV (PTV-GTV) and CTV (PTV-CTV) covered the GTV and CTV with a 0.5 cm margin (10), respectively. SMART technique was used, and treatment plans were generated by the Monaco treatment planning system (Elekta). Radiation was delivered with 6-MV photons by a linear accelerator. The prescribed doses were 64 Gy for PTV-GTV (2.1 Gy/fraction) and 48 Gy for PTV-CTV (1.6 Gy/fraction) in 30 fractions. It was required that 95% of the PTV receive the prescribed dose. Dose constraints for normal structures included: mean lung dose <20 Gy and the total lung volumes irradiated above 20 Gy (V20) <30%; V40 of the heart <30%; maximum dose of spinal cord dose ≤45 Gy; D0.5cc of the small bowl ≤45 Gy; maximum dose of the stomach <54 Gy; and V18 of the kidney <30%. In case of grade 4 myelosuppression, or ≥grade 3 nonhematologic toxicities that lasted longer than one week, RT was stopped until the toxicities resolved to ≤grade 2. For patients with a break ≥2 weeks, a new plan for a dose boost to PTV-GTV would be given at clinical discretion.

CDDP (25mg/m2) was delivered intravenously on day 1 of each week of RT, and S1 (40 mg/m2, bid) was delivered orally on days 1–14 and 22–35 during RT. For patients who could not swallow S1 capsule, the powder of S1 would be administered through the tube. Chemotherapy administration could be interrupted in case of adverse effects. Then a dose adjustment on weekly basis was needed when the adverse effects resolved.

Routine nutritional support was performed from the start of CCRT, including oral nutritional supplements, enteral nutrition *via* nasogastric tube or percutaneous endoscopic gastrostomy, and/or parenteral nutrition.

### Evaluation

Patient history, physical examination, complete blood count, serum chemistries, endoscopy, chest/upper abdomen CT, chest magnetic resonance imaging (MRI), bone scan and/or PET scan were obtained before CCRT. Nutritional risk index (NRI) was calculated as: 1.519 × serum albumin level (g/L) + 41.7 × (present/usual weight). Neutrophil-lymphocyte ratio (NLR) was calculated as: the absolute neutrophils count/the absolute lymphocyte count. Charlson score was used for the evaluation of comorbid condition (16). Complete blood count (CBC) and serum chemistries were obtained weekly during CCRT. Objective response was assessed by endoscopy, chest/upper abdomen CT and chest MRI two months after CCRT according the tri-modality criteria (17). Assessment of disease by endoscopy, chest/upper abdomen CT and chest MR were first performed two months after CCRT, and then every 3-4 months for the first 2 years, every 6 months for years 3 to 5, and yearly thereafter. Bone scan or PET scan were performed when the patient was suspected for distant progression. Treatment related toxicities were graded by the Common Terminology Criteria for Adverse Events version 4.0 (CTCAE 4.0) from the start of radiotherapy until 2 months afterward. In particular, pneumonitis was observed from the start until one year after radiotherapy. The maximum observable toxicities were recorded.

### Statistical Analysis

The primary endpoint of this study was objective response rate (ORR). ORR was defined as the percentage of patients who achieved partial or complete remission two months after CCRT (17). We assumed that the ORR could be improved from 60% according to previous published data to 80% in the current study. Enrollment of 36 patients was required to yield 80% power to detect an expected improvement based on a one-sided 0.025 level test. Considering the rate of dropout as 10%, planned enrollment was 40 patients.

The secondary endpoints included overall survival (OS), progression-free survival (PFS), loco-regional recurrence-free survival (LRFS), distant metastasis-free survival (DMFS) and toxicities. Endpoints of OS, locoregional recurrence and distant metastasis were measured from the start of CCRT. Correlation between clinical variables and tumor response was performed by the Chi-square test. Survival analyses were performed using the Kaplan-Meier method. Correlation between clinical variables and survival was performed using Cox proportional hazards model. Variables with a p-value <0.05 in univariate analysis were included in the multivariate model. The statistical analysis was performed using SPSS 24.0. A p-value <0.05 was considered statistically significant.

## Results

### Patients

**Figure 1:**
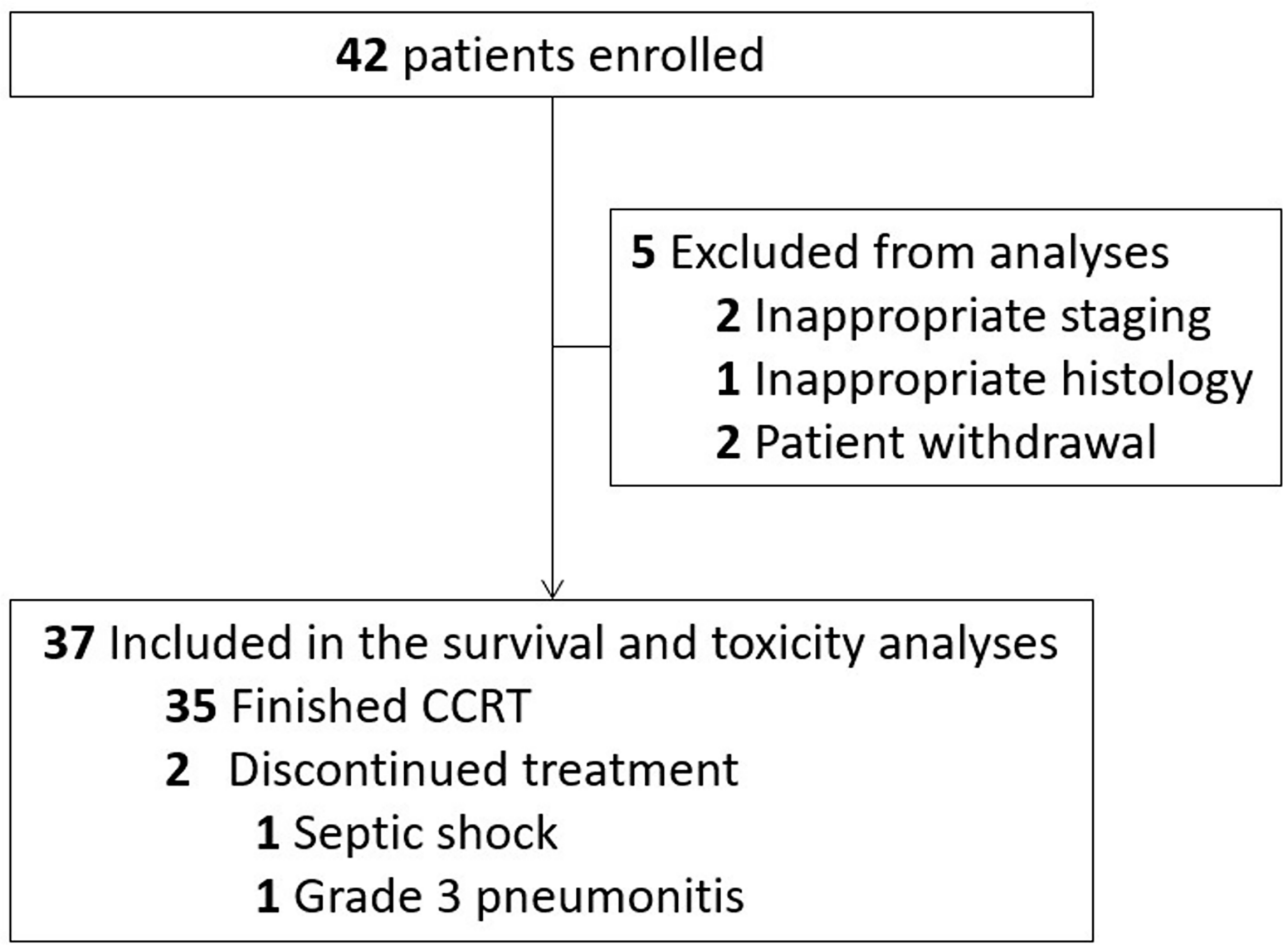
Trial profile. CCRT, Concurrent chemoradiotherapy.

Between July 2015 and June 2018, 42 patients with stage II–IVa ESCC were enrolled in this study. Five patients were excluded from analyses because of distant metastasis before treatment (n=2), inappropriate histology (n=1) or patient withdrawal (n=2). Thirty-seven patients were included in the current analyses (**Figure 1**). The characteristics of the analyzed patients are detailed in **Table 1**. At the time of last follow-up (June 20, 2020), 16 patients (43.2%) were alive and 21 patients (56.8%) were dead. Median follow-up time was 25.7 months (range, 1.1-59.0 months) for all and 46.1 months (range, 19.5-59.0 months) for living patients.

**Table 1:** Baseline characteristics (n = 37).

Characteristics	n (%)
Age (years)	
Median (Range)	73 (70–77)
Sex	
Female	10 (27.0)
Male	27 (73.0)
ECOG performance status	
0-1	35 (94.6)
2	2 (5.4)
Charlson score	
0	28 (75.7)
1	6 (16.2)
2	2 (5.4)
3	1 (2.7)
Percent weight loss at diagnosis^#^	
<5%	25 (67.6)
≥5%	12 (32.4)
Primary tumor location	
Cervical	4 (10.8)
Proximal third	8 (21.6)
Middle third	21 (56.8)
Distal third	3 (8.1)
Multiple origin	1 (2.7)
Primary tumor length (mm)*	
Median (Range)	58 (12–125)
cTNM stage	
II	10 (27.0)
III	21 (56.8)
IVa	6 (16.2)
GTV volume (cm^3^)	
Median (Range)	60.5 (7.5-176.8)
Baseline NRI	
Median (Range)	105.2 (95.4-111.6)
Baseline NLR	
Median (Range)	2.56 (1.10-5.42)
Baseline HGB	
Median (Range)	129 (98–152)
Baseline CRP	
<10mg/L	27 (73.0)
≥10mg/L	10 (27.0)

ECOG PS, Eastern Cooperative Oncology Group (ECOG) performance status; GTV, gross tumor volume; NRI, nutritional risk index; NLR, neutrophil-lymphocyte ratio; HGB, hemoglobin; CRP, C-reactive protein. ^#^Percent weight loss at diagnosis was defined as the percentage of weight loss in the past three months before diagnosis (18). *Primary tumor length was the endoscopically measured tumor length.

### Treatment Compliance

Treatment compliance is detailed in **Table 2**. Of the 37 patients, 22 patients (59.5%) completed the planned RT as planned. Other than that, there were 13 patients (35.1%) who completed RT with a break ≥7days due to persistent grade 3 esophagitis (n=6), grade 3 fatigue (n=5) or weight loss ≥10% during treatment (n=2). Two patients (5.4%) discontinued treatment and received a radiation dose < 50 Gy due to grade 5 sepsis (n=1) or grade 3 pneumonitis (n=1). The median treatment duration was 43 days (range, 39-134 days) for those who completed RT.

**Table 2:** Treatment compliance (n = 37).

RT compliance (n, %)	
Completion of RT as planned	22 (59.5)
Completion of RT with break of 7–80 days	12 (32.4)
Completion of RT with break ≥80 days	1 (2.7%)
Discontinue RT	2 (5.4)
RT dose received by PTV-GTV (n, %)	
64 Gy	35 (84.6)
<64 Gy	2 (5.4)
RT durations (days, n = 35)	
Median	43
Range	39-134
CDDP delivery (weeks)	
2	7 (18.9)
3	7 (18.9)
4	18 (48.7)
5	4 (10.8)
6	1 (2.7)
S1 delivery (weeks)	
1	1 (2.7)
2	5 (13.5)
3	4 (10.8)
4	27 (73.0)
Enteral nutrition	
Oral supplements	21 (56.8%)
Nasogastric tube	7 (18.9%)
Percutaneous endoscopic gastrostomy	9 (24.3%)

RT, radiotherapy.

Thirty-two patients (86.5%) completed ≥4 weeks of CDDP, and 27 (73.0%) completed 4 weeks of S1. The reasons for dose modification included myelosuppression (thrombocytopenia in 4 patients, neutropenia in 3 patients and both in 1 patient), gastrointestinal toxicities (n=5) and decline in nutrition status (n=2).

Enteral nutrition during CCRT was performed *via* oral supplements, nasogastric tube and percutaneous endoscopic gastrostomy in 21 (56.8%), 7 (18.9%) and 9 (24.3%) patients respectively.

### Response to CCRT and Survival

Thirty-six (36/37) patients were assessed for response two months after the end of CCRT (one patient died during CRT due to septic shock). There were 22 (59.5%) with complete remission (CR) of disease, 10 (27.0%) with partial remission (12) and 4 (10.8%) with progressive disease (PD). Progressive disease occurred in distant sites in three patient and in locoregional site in one patient. The objective response (CR+PR) rate was 88.9% (32/36). Gross tumor volume change two months after the therapy is shown in **Supplementary Figure 1**. Gross tumor reduction >70% was achieved in all patients with PR. The correlation between clinical variables and tumor response was explored (**Table 3**). Patients with baseline weight loss <5% (p=0.050) and baseline NRI ≥105.2 (p=0.023) tended to have better tumor response two months after CCRT.

**Table 3:** Correlation between tumor response and clinical variables (n = 36).

Variables	p value
Sex (male *vs*. female)	0.301
Age (≥73 *vs*. <73 yrs)	0.318
ECOG performance status (2 *vs*. 0-1)	0.263
Charlson score (0-1 *vs*. 2-3)	0.304
Percent weight loss at diagnosis *(≥5% *vs*. <5%)	**0.050**
Stage (III, IVa *vs*. II)	0.056
GTV volume (≥60.5 *vs*. <60.5cm^3^)	0.067
Baseline NRI (≥105.2 *vs*. <105.2)	**0.023**
Baseline NLR (≥2.56 *vs*. <2.56)	0.497
Baseline HGB (≥129 *vs*. <129g/L)	0.478
Baseline CRP (≥10 *vs*. <10mg/L)	0.908
Completion of RT (Completion of RT as planned *vs*. completion of RT with break ≥7days *vs*. Discontinue RT)	0.554

ECOG PS, Eastern Cooperative Oncology Group (ECOG) performance status; GTV, gross tumor volume; NRI, nutritional risk index; NLR, neutrophil-lymphocyte ratio; HGB, hemoglobin; CRP, C-reactive protein. *Percent weight loss at diagnosis was defined as the percentage of weight loss in the past three months before diagnosis (18).

The bold values mean these p-values are statistically significant (p<0.05).

**Figure 2:**
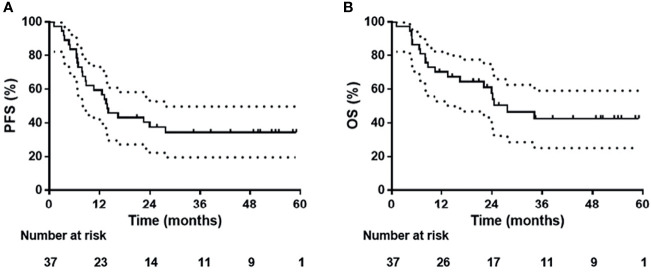
**(A)** Progression-free survival and **(B)** overall survival curves for 37 patients.

Twenty-four (64.9%) of 37 patients had disease progression or died at last follow-up. Median PFS was 13.8 months (95% CI, 9.3-18.4 months), with 1-year, 2-year and 3-year PFS rates of 59.5% (95% CI, 43.6-75.4%), 37.5% (95% CI, 21.8-53.2%) and 34.4% (95% CI, 18.9-49.9%), respectively (**Figure 2A**). Twenty-one (56.8%) died at last follow-up. The estimated median OS was 27.7 months (95% CI, 15.8-39.7 months), with 1-year, 2-year and 3-year OS rates of 70.3% (95% CI, 55.6-85.0%), 57.5% (95% CI, 40.8-74.2%) and 42.6% (95% CI, 25.0-60.2%), respectively (**Figure 2B**).

**Figure 3:**
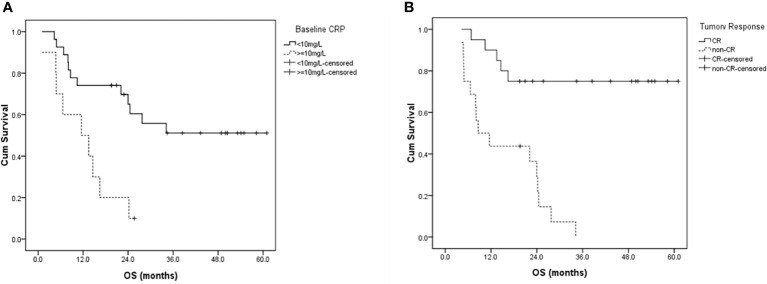
Overall survival curves for patients with **(A)** different baseline CRP levels, and **(B)** different tumor responses two months after radiotherapy. CRP, C-reactive protein; CR, complete response; OS, overall survival.

As shown in **Table 4**, in univariable analysis, median OS was significantly correlated with ECOG performance score (2 *vs* 0-1, 1.1 *vs* 27.7 months, p=0.005), stage (III-IVa *vs* II, 22.1 months *vs* not reached [NR], p=0.014), pre-treatment GTV volume (≥60.5 *vs* <60.5 cm3, 16.5 months *vs* NR, p=0.004), baseline NRI (≥105.2 *vs* <105.2, 16.5 months *vs* NR, p=0.036), baseline CRP level (≥10 *vs* <10mg/L, 13.5 months *vs* NR, p=0.003) and tumor response (non-CR *vs* CR, 13.5 months *vs* NR, p=0.000). OS showed no significant difference between patients who completed RT as planned and those who completed RT with break ≥7 days (27.7 *vs* 34.1 months, p=0.787). In multivariable analysis, baseline CRP level (p=0.016) and tumor response (p=0.021) were independently prognostic of OS (**Figure 3**).

**Table 4:** Univariable and multivariable analysis of prognostic factors for overall survival.

Variables	Univariable analysis		Multivariable analysis	
	HR, 95% CI	p value	HR, 95% CI	p value
Sex (male *vs*. female)	3.376, (0.980-11.626)	0.054		
Age (≥73 *vs*. <73 yrs)	1.028, (0.414-2.549)	0.953		
ECOG performance status (2 *vs*. 0-1)	9.774, (1.958-48.801)	**0.005**	4.036, (0.365-44.661)	0.255
Charlson score (0-1 *vs*. 2-3)	0.506, (0.067-3.796)	0.508		
Percent weight loss at diagnosis* (≥5% *vs*. <5%)	2.206 (0.920-5.287)	0.076		
Stage (III, IVa *vs*. II)	12.555, (1.669-94.463)	**0.014**	3.977, (0.398-39.734)	0.240
GTV volume (≥60.5 *vs*. <60.5cm^3^)	4.022, (1.547-10.460)	**0.004**	1.149, (0.352-3.746)	0.818
Baseline NRI (≥105.2 *vs*. <105.2)	0.377, (0.151-0.938)	**0.036**	0.918, (0.252-3.345)	0.936
Baseline NLR (≥2.56 *vs*. <2.56)	1.157, (0.490-2.732)	0.739		
Baseline HGB (≥129 *vs*. <129g/L)	1.318, (0.553-3.137)	0.533		
Baseline CRP (≥10 *vs*. <10mg/L)	3.981, (1.588-9.977)	**0.003**	1.020, (1.004-1.037)	**0.016**
Completion of RT (Completion of RT as planned *vs*. completion of RT with break ≥7days)	1.143, (0.435-3.005)	0.787		
Tumor response (non-CR *vs*. CR)	6.632, (2.978-14.772)	**0.000**	4.088, (1.236-13.518)	**0.021**

ECOG PS, Eastern Cooperative Oncology Group (ECOG) performance status; GTV, gross tumor volume; NRI, nutritional risk index; NLR, neutrophil-lymphocyte ratio; HGB, hemoglobin; CRP, C-reactive protein. *Percent weight loss at diagnosis was defined as the percentage of weight loss in the past three months before diagnosis (18).

The bold values mean these p-values are statistically significant (p < 0.05).

### Failure Patterns

At the time of analysis, 8 patients (21.6%) developed loco-regional recurrence. 2-year LRFS was 64.4% (95%CI, 44.2-84.6%). Thirteen patients (35.1%) developed distant metastasis. 2-year DMFS was 59.1% (95%CI, 40.7-77.5%). The failure pattern is detailed in **Supplementary Figure 2**. Distant metastasis was the main cause of treatment failure with lungs being the most common involved site.

### Toxicities

Treatment related toxicities are listed in **Table 5**. ≥Grade 3 hematologic toxicities included anemia in 1 (2.7%) patient, neutropenia in 4 (10.8%) patients and thrombocytopenia in 5 (13.5%) patients. Grade 3 non-hematologic toxicities included esophagitis in 7 (18.9%) patients, pneumonitis in 1 (2.7%) patient, gastrointestinal toxicity in 1 (2.7%) patient, fatigue in 1 (2.7%) patient and bleeding in 1 (2.7%) patient. No grade 4 non-hematologic toxicities were developed. Grade 5 sepsis occurred in 1 (2.7%) patient.

**Table 5:** Treatment related toxicities (n = 37).

Toxicity	Grade, No. (n/37%)				
	1	2	3	4	5
Non-hematologic					
Esophagitis	10 (27.0)	20 (54.1)	7 (18.9)	0	0
Pneumonitis	24 (64.9)	2 (5.4)	1 (2.7)	0	0
Gastrointestinal	14 (37.8)	9 (24.3)	1 (2.7)	0	0
Arrhythmia	2 (5.4)	1 (2.7)	0	0	0
Fatigue	7 (18.9)	3 (8.1)	1 (2.7)	0	0
Skin	8 (21.6)	3 (8.1)	0	0	0
Weight loss	3 (8.1)	1 (2.7)	0	0	0
Bleeding	2 (5.4)	0	1 (2.7)	0	0
Sepsis	0	0	0	0	1 (2.7)
Hematologic					
Anemia	12 (32.4)	18 (48.6)	1 (2.7)	0	0
Neutropenia	6 (16.2)	8 (21.6)	3 (8.1)	1 (2.7)	0
Thrombocytopenia	7 (18.9)	10 (27.0)	2 (5.4)	3 (8.1)	0
ALT elevation	3 (8.1)	0	0	0	0
AST elevation	2 (5.4)	0	0	0	0
Creatinine elevation	1 (2.7)	2 (5.4)	0	0	0

Toxicities were graded by CTCAE version 4.0.

ALT, alanine aminotransferase; AST, aspartate aminotransferase.

## Discussion

The treatment for ESCC in elderly patients remains challenging due to the decreased physiologic reserve, increased prevalence of cardiopulmonary comorbidities, and increased risk of treatment-related toxicities in this population. The current study prospectively assessed the efficacy and toxicity of SMART concurrently with CDDP/S1 in 37 elderly patients with ESCC. Thirty-five (35/37, 94.6%) patients completed the SMART, while approximately one third of them experienced a treatment break ≥7days. The ORR was 88.9%, beyond the assumption goal of 60%. The median OS and PFS was 27.7 and 13.8 months respectively. Toxicities were acceptable with ≥grade 3 esophagitis in 7 (18.9%) patients and pneumonitis in 1 (2.7%) patient. Grade 4 side effects included neutropenia in 1 (2.7%) patient and thrombocytopenia in 3 (8.1%) patients. Treatment-related death occurred in 1 (2.7%) patient due to septic shock.

Some studies have evaluated the efficacy and safety of definitive CCRT in elderly patients and indicated that CCRT was a feasible strategy (5–8, 19–24). More information details were shown in **Table 6**. These studies delivered RT at doses ranging from 50 to 60Gy. The RT technique included 2D, 3D and IMRT. Concurrent chemotherapy regimen included CDDP/carboplatin plus 5-fluorouracil (5-FU), CDDP plus paclitaxel, CDDP plus capecitabine and single-agent regimen. The ORR ranged from to 56.7 to 84%. The median OS ranged from 9 to 35 months, with 2-year OS rate of 27 to 78%. Small sample size, different inclusion criteria, different RT technique/dose, and diverse chemotherapy regimen might account for the difference in survival outcomes. To the best of our knowledge, our study is the first prospective study assessing SMART concurrently with CDDP/S1 in elderly patients. Wang et al. retrospectively evaluated the feasibility and efficacy of CCRT with CDDP/S1 for elderly ESCC patients (21). The radiation dose was lower than ours (54 *vs.* 64 Gy). The chemotherapy regimens were similar except that CDDP was delivered as a three-months manner in their study. We achieved a higher ORR (88.9 *vs.* 84.0%) and OS (27.7 *vs*. 18.2 months) possibly due to the higher radiation dose.

**Table 6:** Previous studies of radiotherapy for elderly patients with esophageal cancer.

Study	Study nature	N	Age	Stage	Treatment group	Radiation technique	Radiation therapy dose prescription	Chemotherapy regimens	Median OS (mo)	2-year OS rate (%)	ORR (%)	≥3 grade esophagitis (%)	≥3 grade pneumonitis (%)
Takeuchi (7)	Retrospective	33	≥71	II–III	CCRT	–	60 Gy/30 fractions	CDDP/5-FU	14.7	47*	63.6 (CRR)	9.1	–
Tougeron (5)	Retrospective	109	≥70	I-IV	CCRT	–	50–55 Gy (1.8 or 2 Gy/day)	CDDP/5-FU or CDDP+irinotecan	15.2	35.5	57.8 (CRR)	–	–
Rochigneux (6)	Retrospective	58	≥75	IIB-IIIC	CCRT	3D-CRT	The mean dose was 50.9 Gy (range, 27–72 Gy)	CDDP or CDDP/5-FU or 5-FU	14.5	25.9	–	–	–
Zhang (24)	Retrospective	128	≥65	I-IV	CCRT RT alone	3D-CRT or IMRT	60 Gy (range, 46–70 Gy)/25–35 fractions	Docetaxel+CDDP or CDDP/5-FU	22 13	55* 42*	69.9 47.3	5.5 3.6	2.7 1.8
Servagi‐Vernat (23)	Prospective phase II single-arm study	30	≥75	II-III	CCRT	–	50 Gy/25 fractions	CDDP or oxaliplatin	14.5	28*	73.3	–	–
Li (22)	Retrospective	116	≥70	I-IV	CCRT sCRT RT alone	3D-CRT or IMRT	The median dose was 60Gy (range, 20-70 Gy)/1.8-2Gy per fraction	Docetaxel or CDDP/5-FU or carboplatin+paclitaxel or doxifluridine	22.3 18.0 12.4	50* 38* 30*	- - -	25 16.7 13.3	0 0 0
Song (8)	Retrospective	82	≥70	I-IV	CCRT	3D-CRT	60 Gy/30 fractions	Paclitaxel+CDDP	26.9	–	69.1	8.5	–
Wang (21)	Retrospective	56	≥70	II-IV	CCRT	–	54 Gy/27–30 fractions	CDDP/S1	18.2	44*	84	14.3	3.6
Chen (20)	Retrospective	90	≥65	IIb-III	CCRT RT alone	3D-CRT	56.0–59.4 Gy/30–33 fractions	CDDP/S1	30.6 18.7	78* 20*	73.5 51.2	26.5 14.6	6.1 9.8
Huang (19)	Retrospective	271	≥65	I-IV	RT alone single‐agent‐based CCRT double‐agent‐based CCRT	2D-RT, or 3D-CRT or IMRT	The mean dose was 58.4 ± 6.4 Gy (range, 40‐74 Gy)	Single agents: 5-FU, platinum, and docetaxel Double agents: platinum combined with 5-FU or paclitaxel or docetaxel	15.6 28.8 27.8	39 59 57	60.3 67.2 82.1	20.4 (G2-3) 32.1 (G2-3) 42.7 (G2-3)	0.9 (G2-3) 3.6 (G2-3) 3.2 G2-3)
Our study	Prospective phase II Study	37	70-80	II–IVa	CCRT	SMART	64 Gy/30 fractions	CDDP/S1	27.7	57.5	88.9	18.9	2.7

*Estimating from the survival curve.

3D-CRT, 3D conformal radiotherapy; 5-FU, 5-fluorouracil; CCRT, concurrent chemoradiotherapy; CDDP, cisplatin; CRR, complete response rate; IMRT, intensity modulated radiotherapy; ORR, objective response rate; OS, overall survival; RT, radiotherapy; sCRT, sequential chemoradiotherapy; SMART, simultaneous modulated accelerated radiation therapy.

Despite emerging evidence of CCRT for elderly patients with ESCC, the optimal treatment strategy remains to be elucidated. The first question is the selection of proper concurrent chemotherapeutic drugs. Previous study showed CDDP and 5-FU concurrently with CCRT might not be an appropriate regimen for elderly patients because of frequent treatment discontinuation (57.6%) and substantial grade 3 hematological toxicities (7). S1, an oral fluoropyrimidine, showed several advantages over 5-FU when used as a radiosensitizer (13). It could prolong the half-life of 5-FU in plasma. The oral and daily delivery method shortens hospitalization and makes dose modification convenient. Several studies showed platinum/S1 concurrently with CCRT exhibited encouraging efficacy and manageable toxicity in non-age-selected esophageal cancer, with myelosuppression being the most common adverse effect (14, 15, 25). In a prospective study evaluating CCRT with nedaplatin/S1 in stage II/III esophageal cancer, CR was achieved in 80% of 20 patients, and the 3-year OS was 58.0% (25). Grade 3-4 neutropenia, thrombocytopenia and anemia occurred in 18%, 12% and 6% of patients, respectively. In another phase II study of CCRT with CDDP/S1 in 116 patients with stage II-IVa esophageal cancer, the median PFS and OS were 14.4 and 27.6 months respectively (14). Grade 3-4 neutropenia thrombocytopenia and anemia occurred in 37.9%, 13.8% and 9.5% of patients. The survival data of these studies seemed to be better than that using CCRT concurrent with CDDP/5-FU, with a median OS of 13-17.5 months (3, 26). Based on the above evidence, we chose CDDP/S1 as concurrent regimen in elderly patients. Considering the decreased reserve in this less-fit population, CDDP was delivered in a weekly manner. Compared with the above studies in non-age-selected patients, our study showed similar survival outcomes and hematological toxicities in elderly patients. It is noteworthy that about one third of patients needed chemotherapy dose reduction mostly due to hematological toxicities in our study. The weekly delivered CDDP and daily delivered S1 allowed for in-time modification of drug dose, which was important for elderly patients with decreased bone marrow reserve. The suboptimal compliance to chemotherapy in the current study indicates that a modified chemotherapy regimen, such as single-agent chemotherapy, might be better-tolerated in elderly patients.

The more frequent chemotherapy dose reduction in elderly patients was concerned to affect the response rate and locoregional control. Therefore, intensifying the radiation dose to compensate for the inadequate concurrent drug delivery might be an option to increase treatment efficacy. At the same time, treatment-related toxicities must be considered when escalating RT dose. A population-based analysis included 2553 elderly patients (>65 years) with esophageal cancer treated with either 3-dimensional radiotherapy (3DCRT) or IMRT (27). The use of IMRT was associated with lower cardiac mortality and all-cause mortality compared with 3DCRT. In the current study, we used SMART technique to deliver an escalated dose of 64 Gy to gross tumor with a fraction dose of 2.13 Gy. The relatively high biological effective dose may explain the promising response rate and loco-regional control. Meanwhile, ≥grade 3 pneumonitis occurred in 1 (2.7%) patient and no cardiopulmonary cause death was observed with median follow-up of 25.7 months. These results suggested that dose intensification *via* SMART could be a good choice for the treatment of elderly patients with ESCC, which enables improvement in tumor response and better preservation of organ function. Longer follow up was needed for a better understanding of late toxicities.

General health condition of elderly patients needed special attention before the delivery of CCRT. Nutrition status and systemic inflammatory response have been reported as prognostic factors independent of age, performance status and clinical stage in patients with esophageal cancer (28–30). NRI, calculated by serum albumin and weight, is an objective and simple tool for assessment of nutrition risk. This index has been proposed for the evaluation of nutrition status in patients with various chronic disease (31). Our study showed that patients with baseline weight loss <5% and baseline NRI ≥105.2 tended to have better tumor response two months after CCRT. Baseline NRI was also predictive of OS. This was consistent with the results from non-age-selected population. Inflammation factors were reported to correlate with survival outcome in various cancer types including esophageal cancer (29). We explored the potential role of inflammation-based prognostic factors including CRP and NLR on OS. Baseline CRP level was found to be independently prognostic of OS. These results suggest that the baseline assessment of nutritional and inflammation status using routine clinical variables could predict survival in elderly patients, and serves an important basis for the individualized anti-cancer and supportive therapy. It’s unclear how the dynamic changes of these factors during CCRT influence clinical outcomes and it remains to be further investigated in the future.

As indicated in the multivariate analysis for OS, tumor response two months after CCRT was prognostic of OS. It motivates to assess tumor response as early as possible to adjust the treatment accordingly. Alternative treatment approaches, such as immunotherapy could be investigated for patients that have a poor response to the initial treatment protocol. Advanced disease stage and large GTV volume adversely affects the objective tumor response with marginal significance (**Table 3**). They were also significantly associated with overall survival in univariate analysis. These results were in line with previous studies on esophageal cancer (32, 33).

The analysis of treatment compliance revealed that treatment break was common in CCRT for elderly patients who are more susceptible to treatment toxicities due to the decreased physiologic reserve (8, 21). In our study, about one third of patients had a break ≥7 days during RT due to toxicities. From radiobiologic perspective, prolonging overall treatment time results in decreased tumor control probability and is therefore not desirable. Nevertheless, for elderly patient, a planned treatment break might help reduce treatment-related morbidity and maintain good general condition. Univariable analysis in our cohort showed that delayed and normal timed patients did not show a difference in OS. The relatively high radiation dose compensating for tumor repopulation during the break might explain the result of univariable analysis. It also implies that maintaining good general condition was as important as treatment consistency in this less-fit population.

In conclusion, our study showed that the SMART concurrently with CDDP/S1 yielded satisfactory response rate, survival outcomes and tolerable treatment-related toxicities in elderly patients with ESCC. Baseline CRP and tumor response were prognostic of overall survival. This study was limited by the relatively small number of patients and single-arm design. Randomized studies with larger sample size are warranted to further evaluate the efficacy and toxicity of this treatment approach.

## Data Availability Statement

The raw data supporting the conclusions of this article will be made available by the authors, without undue reservation.

## Ethics Statement

The studies involving human participants were reviewed and approved by Sun Yat-Sen University Cancer Center IRB, Sun Yat-Sen University. The patients/participants provided their written informed consent to participate in this study.

## Author Contributions

Study conception and design: SG, FL, HL, HF, and BQ. Literature review: YW. Data acquisition: YW, FL, SG, XZ, WY, GL, QL, and HF. Statistical analysis: NC, NH, HL, and BW. Data interpretation: NC, JZ, and ML. Manuscript preparation: SG, FL, HL, HF, and BQ. Manuscript review: All authors. All authors contributed to the article and approved the submitted version.

## Conflict of Interest

The authors declare that the research was conducted in the absence of any commercial or financial relationships that could be construed as a potential conflict of interest.

## Publisher’s Note

All claims expressed in this article are solely those of the authors and do not necessarily represent those of their affiliated organizations, or those of the publisher, the editors and the reviewers. Any product that may be evaluated in this article, or claim that may be made by its manufacturer, is not guaranteed or endorsed by the publisher.
