# Supplementary material for: A Prospective Phase II Study of Simultaneous Modulated Accelerated Radiotherapy Concurrently With CDDP/S1 for Esophageal Squamous Cell Carcinoma in the Elderly
Source: Front Oncol. 2021 Nov 25;11:760631. doi: 10.3389/fonc.2021.760631 (PMC8654786; doi:10.3389/fonc.2021.760631)

Supplementary Figure 1. Gross tumor volume change for patients who achieved CR or PR two months after CCRT.

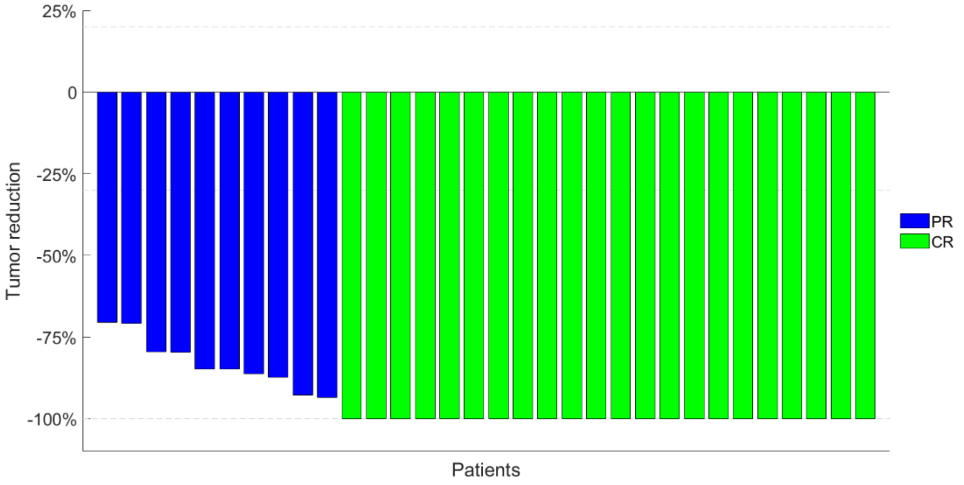

Abbreviations: PR, partial remission; CR, complete remission; CCRT, concurrent radiotherapy.

Supplementary Figure 2. Failure pattern (n=37).

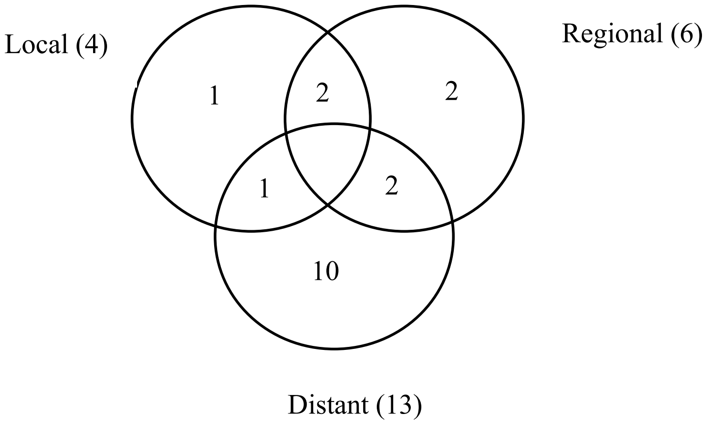

Supplement: Supplementary file 1 [file DataSheet_1.pdf]
